# Vitamin D as an Immuno-Endocrine Modulator: Discovering Its Role in Autoimmune Disorders and Host Defense Mechanisms

**DOI:** 10.3390/jcm15124742

**Published:** 2026-06-18

**Authors:** Sandesh Shende, Jaishriram Rathored

**Affiliations:** Central Research Laboratory and Molecular Diagnostics, School of Allied Health Sciences, Datta Meghe Institute of Higher Education and Research, Sawangi (Meghe), Wardha 442001, Maharashtra, India; sandeshshende.ahs@dmiher.edu.in

**Keywords:** calcium-phosphate homeostasis, skeletal health, immune cells, endocrine cells, gene transcription, cytokine balance, immune tolerance

## Abstract

**Background/Objectives:** Vitamin D, universally recognized for its role in calcium–phosphate homeostasis and skeletal health, has emerged as a key immuno-endocrine modulator. Its active metabolite interacts with the vitamin D receptor (VDR) across immune and endocrine cell populations, influencing gene transcription, cytokine balance, and immune tolerance. This narrative review synthesizes mechanistic, epidemiological, and clinical evidence on the role of vitamin D in immune modulation across autoimmune and infectious diseases. **Methods:** This narrative review incorporated a structured and comprehensive literature search across PubMed/MEDLINE, Scopus, Web of Science, Embase, and Google Scholar. **Results:** Vitamin D modulates both innate and adaptive immunity through antimicrobial peptide induction, macrophage and NK cell activation, and promotion of tolerogenic dendritic cells. Clinical and interventional trial outcomes remain heterogeneous and are influenced by baseline vitamin D status, dosing regimens, genetic variability, and disease context. **Conclusions:** Vitamin D functions in endocrine and immune regulation, contributing to host defense and immune tolerance. Current evidence supports that for autoimmune and infectious conditions, well-designed randomized trials are required to clarify effective dosing, identify responsive subpopulations, and elucidate genetic determinants of therapeutic benefit.

## 1. Introduction

Vitamin D was historically confined to skeletal biology. Over the last two decades, a paradigm shift has established vitamin D as a pleiotropic immuno-endocrine regulator with systemic relevance beyond bone health. Its active metabolite binds the vitamin D receptor (VDR), which is widely expressed in immune and endocrine tissues, enabling regulation of immune tolerance, inflammation, and host defense. The broad tissue distribution of VDR highlights vitamin D’s integrative role in coordinating immune and endocrine signaling rather than acting as a classical micronutrient alone [1]. Considered a fat-soluble secosteroid underlying the calcium-phosphate regulation and skeletal growth, vitamin D has been considered to be the foundation of bone and mineral homeostasis; its physiological role is traditionally confined to prevention of rickets, osteomalacia, and osteoporosis [2]. However, in the past 20 years, a paradigm shift in the scientific community has taken place, bringing light to the discovery of vitamin D as an effective immuno-endocrine modulator that has widespread systemic effects [3]. Its active form, 1,25-dihydroxyvitamin D (1,25(OH)_2_D_3_ or calcitriol), binds to the nuclear VDR, a widely distributed receptor on immune cells, secretory endocrine glands, and epithelial barriers, thus exerting far more extensive effects than those on the musculoskeletal system [4]. The omnipresent presence of vitamin D in the various tissues highlights its critical importance as a molecular connector, uniting the immune system and endocrine balance [5]. Studies on vitamin D have increased tremendously since the vitamin D receptor VDR has been identified in the whole immune cell population, including macrophages, dendritic cells (DCs), T cells, and B cells [6]. The binding to the vitamin D response element (VDRE) in promoter sequences of certain genes is triggered by VDR engagement and heterodimerization with the retinoid X receptor (RXR) [7]. These molecular actions put vitamin D at the border of immunology and endocrinology, giving it the ability to regulate both innate and adaptive responses [8]. It is a genomic pathway that influences genes (over nine hundred genes) that deal with immunity, cell proliferation, apoptosis, and cytokines generation [9]. Simultaneously, quick signaling with non-genomic effects helps in the propagation of the biological impacts of vitamin D [10].

Vitamin D was historically confined to skeletal biology, primarily associated with calcium–phosphate homeostasis and the prevention of rickets, osteomalacia, and osteoporosis [11]. Over the past two decades, a paradigm shift has established vitamin D as a pleiotropic immuno-endocrine regulator with systemic relevance extending far beyond bone health. Its hormonally active metabolite, calcitriol, binds to the vitamin D receptor (VDR), which is widely expressed in immune cells, endocrine tissues, and epithelial barriers [2]. The broad tissue distribution of VDR highlights vitamin D’s integrative role in coordinating immune and endocrine signaling rather than functioning solely as a classical micronutrient. VDR activation triggers heterodimerization with the RXR and binding to VDREs, regulating the transcription of hundreds of genes involved in immunity, cellular proliferation, apoptosis, and cytokine production [12]. In addition to genomic effects, vitamin D exerts rapid non-genomic and epigenetic actions that further modulate immune responsiveness. Accumulating evidence indicates that hypovitaminosis D is common in autoimmune disorders such as multiple sclerosis (MS), type 1 diabetes mellitus (T1DM), rheumatoid arthritis (RA), and systemic lupus erythematosus (SLE), as well as in infectious diseases, including tuberculosis and acute respiratory tract infections [13]. While mechanistic and observational data strongly support an immunomodulatory role for vitamin D, translation into consistent clinical benefit remains challenging, necessitating careful interpretation of evidence and individualized therapeutic strategies [14].

Vitamin D is essential for the functioning of innate immunity, which is the initial immunity that the body possesses [15]. The first immunity that the body has and that is vital in the functioning of the innate immunity is the one that is facilitated by vitamin D. Calcitriol enhances the capability of the organism to eliminate bacterial pathogens and has antiviral protection that enhances the action of antimicrobial (cathelicidin, IL-37) and defensin peptides [16]. It also plays a role in immune homeostasis, enhancing macrophage phagocytosis and chemotaxis, and suppressing an exorbitant production of pro-inflammatory cytokines [17]. The exposure of DCs to vitamin D causes them to adopt a tolerogenic shape, promoting regulatory T cells (Tregs) and reducing antigen presentation [18]. When vitamin D is exposed to DCs, it assumes a tolerogenic shape that favors regulatory T cells (Tregs) and decreases antigen presentation. In addition to that, vitamin D enhances the activity of NK cells, thereby boosting the production of interferon-γ and the viral cytotoxic mechanism. All these findings could be utilized to demonstrate how vitamin D, at the same time, induces immunological tolerance and enhances antimicrobial defense [19].

The levels of vitamin D have a modulatory effect on adaptive immunity. The pro-inflammatory Th1/Th17 lymphocyte subsets involved in autoimmune pathology are inhibited by the activity of the active metabolite calcitriol through the release of cytokines, including IL-12, IL-17, and interferon-γ [20]. On the other hand, vitamin D enhances Th2 cell differentiation and increases T reg cell numbers, thus increasing the generation of anti-inflammatory factors such as IL-10 and transforming growth factor-β [21]. Vitamin D also has a direct effect on the biology of B cells: vitamin D suppresses B-cell growth and prevents differentiation of plasma cells and antibody secretion, the latter of which is particularly important in the case of autoimmune diseases such as systemic lupus erythematosus [22]. Overall, vitamin D coordinates the action of T and B cell compartments in such a way that the homeostasis of the immune system and the elimination of the potential threat of excessive or inappropriate inflammatory reactions are preserved [23].

Hypovitaminosis is chronic in most autoimmune diseases, including multiple sclerosis, type 1 diabetes, rheumatoid arthritis, and systemic lupus erythematosus, which suggests that patients with serum 25(OH)D levels below the optimal range are more susceptible to the onset of these disorders and highlights the modulatory role of vitamin D on immunity [13]. Beyond autoimmunity, the current literature reveals that poor levels of vitamin D status increase vulnerability to a range of pathogenic conditions, in particular, to SARS-CoV-2 infection, lower respiratory tract infections, and tuberculosis [24]. Even though vitamin D deficiency is not the most determining etiological factor, it is more likely to signify an unfavorable health setting, which predisposes patients to severe disease progression. The connection between vitamin D and complications of diseases is another complication that is associated with genetic polymorphism in GC, CYP27B1, and VDR loci. It is these genes that are diverse, which influences the bioavailability of vitamin D, vitamin D activation, and signal transduction triggered by the receptor and hence modifies the individual response to vitamin D, be it endogenously or exogenously supplied [25].

Lowering the daily doses to moderate levels is likely to yield better results in comparison to high doses that lead to adverse effects [26]. Such an outcome trend explains why personalized medicine is crucial. It requires the examination of baseline vitamin D status, specific pathological situations, and the genetic composition of a person [27]. The review describes the molecular immunomodulatory action of calcitriol, the hormonally active metabolite of vitamin D. It also takes into account the debates surrounding vitamin D supplementation as well as its potential for treating autoimmune and infectious diseases. Moreover, vitamin D functions as both a hormone and an immune regulator. It determines pathogenesis, general predisposition to illness, and susceptibility to diseases. It is situated at the intersection of immunology and endocrinology.

## 2. Materials and Methods

This study was designed as a narrative review to critically synthesize existing evidence on vitamin D as an immuno-endocrine modulator and its role in autoimmune disorders and host defense mechanisms. A narrative approach was selected to allow integrative interpretation of heterogeneous mechanistic, epidemiological, and clinical literature, which is not amenable to quantitative meta-analysis due to variability in study designs, populations, outcomes, and intervention protocols.

A comprehensive literature search was conducted across multiple electronic databases, including PubMed/MEDLINE, Scopus, Web of Science, Embase, and Google Scholar. The search covered publications from January 2000 to March 2025, with the most recent update performed prior to manuscript submission to ensure inclusion of newly published evidence. Search strategies were developed using a combination of Medical Subject Headings (MeSH) and free-text keywords, applied with Boolean operators (AND, OR), truncation, and phrase-based searching. Core search terms included vitamin D, 25-hydroxyvitamin D, 1,25-dihydroxyvitamin D, vitamin D receptor (VDR), immune modulation, innate immunity, adaptive immunity, autoimmune diseases, and infections. Database-specific search adaptations were applied where necessary to optimize retrieval sensitivity. Eligible literature comprised peer-reviewed mechanistic studies (in vitro and in vivo), observational studies, randomized controlled trials, and relevant narrative reviews and meta-analyses published in English. Studies focusing exclusively on skeletal or mineral metabolism without immunological relevance were excluded, as were editorials, commentaries, and non-peer-reviewed reports.

Given the substantial clinical and methodological heterogeneity across included studies, particularly in relation to disease phenotypes, baseline vitamin D status, dosing regimens, genetic polymorphisms (e.g., VDR, CYP2R1, GC), and outcome measures, a narrative synthesis was undertaken. Evidence was organized thematically, with emphasis on molecular and cellular immunology, autoimmune disease mechanisms, infectious disease outcomes, and sources of inconsistency in clinical trial findings. This approach enabled a balanced interpretation of mechanistic plausibility alongside real-world clinical evidence, while avoiding inappropriate causal inference.

## 3. Molecular Basis of Vitamin D Signaling in Immunity

VDR acts as a molecular switch, which conveys the influence of vitamin D in the organism [28]. It is a member of the nuclear receptor superfamily [29]. The activation takes place when VDR interacts with the ligand 1,25-dihydroxyvitamin D3, followed by the heterodimerization of VDR with RXR and translocation to the nucleus [30]. It is there that the complex co-purifies with elements of VDREs and thus regulates the transcription of over 900 genes of interest in immune regulation, apoptosis, and cell-cycle regulation [31]. Other antimicrobial peptides (LL-37 and the body’s first line of defensins) that are part of the innate immune response against bacterial and viral infections are also VDR-regulated genes [32]. VDR signaling cascade also controls the production of cytokines, inhibiting pathogenic inflammatory mechanisms and enhancing anti-inflammatory processes, which maintain host integrity [33]. VDR functions as a ligand-activated transcription factor and molecular switch, integrating immune and endocrine regulation. Upon binding calcitriol, VDR heterodimerizes with RXR and translocates to the nucleus, where it regulates transcription of more than 900 genes implicated in immune homeostasis, apoptosis, and cell-cycle control [34]. VDR signaling induces antimicrobial peptides such as cathelicidin (LL-37) and defensins, which form critical components of innate immunity [35]. Beyond classical genomic effects, vitamin D signaling involves epigenetic remodeling, microRNA regulation, and rapid non-genomic pathways, including MAPK, PI3K, and NF-κB signaling cascades. Genetic polymorphisms in VDR and vitamin D-metabolizing enzymes further contribute to inter-individual variability in immune responsiveness [36].

Vitamin D signaling within the adaptive immune compartment impacts differentiation of tolerogenic DCs and regulatory T cells, which protects self-tolerance and alleviates the autoimmune pathology [37]. It, at the same time, inhibits pro-inflammatory Th1 and Th17 phenotype differentiation. These two effects highlight the critical role of VDR in the exchange of innate and adaptive immune responses, which is of great therapeutic interest [37].

One important aspect of vitamin D signaling is that it communicates with the endocrine axis and the immune system, allowing cells to combine improved metabolic control with immune homeostasis [38]. In the endocrine tissues, VDR is expressed in the thyroid and parathyroid, adrenal glands, and pancreatic β-cells, which provides the systemic action beyond immune modulation. Further, inflammation in itself also regulates the metabolism of vitamin D [39]. CYP27B1 hydroxylase, which converts circulating 25-hydroxyvitamin D into active 1,25(OH)_2_D_3_ in inflamed tissues, is the hydroxylase that becomes activated in the macrophages and DCs [39]. This regionalized conversion allows tightly acting autocrine and paracrine immunoregulatory responses. In tuberculosis, cathelicidin is upregulated by macrophage-generated CYP27B1 and allows killing of mycobacteria in the cell [40]. However, continuous immune activation accelerates localized vitamin D loss, raising the risk of systemic insufficiency and disrupting endocrine homeostasis. In a similar vein, vitamin D has an antagonistic relationship with the endocrine and immune systems [41].

Vitamin D signaling employs microRNA-mediated networks, rapid non-genomic responses, and epigenetic remodeling in addition to canonical transcriptional control to enhance immune function [42]. VDR recruitment of chromatin remodelers modulates the accessibility of loci regulating immunity related to individual differences due to polymorphisms in the VDR gene and its cofactors, altering DNA methylation and histone acetylation and explaining inter-individual variation in responsiveness [43]. The non-genomic signaling induced by VDR, which is membrane-bound or by secondary messengers, instantaneously triggers the signaling of MAPK, PI3K, and NF-κB cascades in response to cytokine regulation and cell survival [44]. Moreover, vitamin D acts in the regulation of microRNAs to control the differentiation of T cells, macrophage activation, and antiviral modes [45]. Vitamin D signaling via VDR regulates transcription of immune-related genes, including antimicrobial peptides and cytokine modulators. Epigenetic remodeling, non-genomic signaling, and microRNA regulation further explain inter-individual variability in immune responsiveness to vitamin D [44]. These orchestrations of molecules demonstrate that vitamin D is a pleiotropic immuno-endocrine regulator and not just a nutrient of the skeleton [46]. The complex clinical effects of vitamin D deficiency and the potential benefits of treating autoimmune disease, infection, and chronic inflammation are explained by a thorough review of these mechanisms.

### 3.1. Vitamin D and Innate Immunity

The innate immune system is the first line of defense against invasive pathogens, and there is growing evidence that vitamin D has profound regulatory effects on this aspect of the host immune system [47]. The regulation of antimicrobial peptides can be regarded as one of the most extensively studied mechanisms. The VDR in the innate immune cells is bound by the active metabolite of calcitriol, which leads to expression of genes that encode the antimicrobial peptides like LL-37 and B-defensins [48]. These peptides have broad-spectrum antimicrobial actions that act through disrupting bacterial membranes, neutralizing endotoxins, and preventing viral replication. Cathelicidin has specifically been identified in mucosal immunity of the respiratory tract, whereby it stimulates the function of the epithelial barrier and restricts viral access [49]. This mechanistic pathway can be used to explain clinical associations of vitamin D deficiency and low prevalence rates of tuberculosis, in which cathelicidin expression mediates macrophage-mediated killing of Mycobacterium tuberculosis [50]. Similarly, vitamin D supplementation has been linked to both the risk and severity of acute respiratory tract infections as well as full defense against a variety of viral pathogens, including influenza and SARS-CoV [51,52]. Interestingly, these peptides are immunomodulators in and of themselves, balancing innate and adaptive responses by attracting immune cells to the infection site and simultaneously stimulating and suppressing excessive tissue degradation. This suggests that vitamin D is both an antimicrobial enhancer and an inflammation regulator [53].

Vitamin D exerts profound effects on innate immunity by enhancing antimicrobial peptide production, macrophage phagocytosis, dendritic cell tolerogenicity, and NK cell cytotoxicity. Local conversion of circulating 25(OH)D to calcitriol by immune cells via CYP27B1 enables autocrine and paracrine immune regulation, particularly at sites of infection [19]. These mechanisms are especially relevant in tuberculosis and respiratory infections, where vitamin D enhances intracellular pathogen clearance. However, clinical translation remains variable and depends on baseline deficiency, dosing strategy, and host genetic factors [54]. Besides the induction of antimicrobial peptides, vitamin D also regulates the work of antigen-presenting and phagocytic cells, especially that of the macrophages and DCs (DCs), which are the center of innate immune responses [46]. Both VDR and the ‘active’ form of the enzyme CYP27B1 are expressed by macrophages, and they can locally convert circulating 25(OH)D to its active state [55]. This autocrine-paracrine pathway augments macrophage chemotaxis, phagocytosis, and bacterial clearance and restrains undue secretion of pro-inflammatory cytokines, including TNF-α, IL-6, and IL-1β [56]. During tuberculosis, public domain activation of macrophages via vitamin D induces fusion of phagolysosomes and discursion of the pathogen, which highlights its applicability in the management of granulomatous diseases [57,58]. Vitamin D, on the other hand, promotes a tolerogenic phenotype in DCs by restraining their maturation, suppressing MHC class II and co-stimulatory molecule expression, and encouraging the release of IL-10 over IL-12 [59]. This bias to an infantile, anti-inflammatory position supports immune tolerance and suppresses pathological responses of T-helper cells, which is especially important to achieve autoimmunity prevention and chronic inflammation [60,61]. This paradoxical, dual regulation, in which vitamin D helps in pathogen clearance but does not activate immune cells too much, makes vitamin D the key control of immune homeostasis [55]. However, there is evidence suggesting that in chronic inflammatory diseases, DCs have decreased sensitivity to vitamin D, which may be a context-dependent effect and is unlikely to be resolved soon in clinical immunology [44].

Vitamin D enhances innate immune defense by inducing cathelicidin and defensins, modulating macrophage phagocytosis, and improving NK cell cytotoxicity. These effects are particularly relevant in respiratory infections and tuberculosis but show variable clinical translation depending on baseline deficiency and dosing strategy [62]. Vitamin D also has a profound effect on NK cell activity, an important part of antiviral defense, as well as its effect on macrophages and DCs [63]. There is evidence that an adequate level of vitamin D improves NK cell cytotoxicity, perforin release, and interferon-7 secretion, which protects against viral elimination [64]. These effects have been found to have specific significance in viral infections, in which NK cell performance is a primary outcome in determining pathology, as it occurs in hepatitis C and respiratory viral diseases [65,66]. Vitamin D, by enhancing NK response, can both promote the rapid elimination of virus using innate immunity and the cytokine environment in a manner that facilitates adaptive polarization of T cells [67]. Observational studies also indicate that a deficiency in vitamin D leads to compromised NK cell activity, which also gives extra credit to the idea that vitamin D is necessary to perform successful innate antiviral surveillance [68,69]. Nevertheless, there are still certain discrepancies, whose elements are interindividual variation in VDR polymorphisms, baseline vitamin D status, and disease setting to determine the extent of NK responsiveness to supplementation [70]. Combined, these data point to the idea that vitamin D strengthens innate immunity on numerous countable points: upregulating antimicrobial peptides, modulating the work of the macrophage and the dendritic cell, and increasing the activity of the NK cell, which makes it an essential immuno-endocrine regulator [71]. However, the current debate over ideal dosage schedules, baseline serum levels of sufficiency, and genetic factors that predict responsiveness must be resolved to apply these mechanistic lessons to clinical practice.

### 3.2. Vitamin D and Adaptive Immunity

Adaptive immunity is a highly augmented, antigen-specific immune defense system, which is very important in the long-term protection of the host [72]. However, its dysregulation often forms the basis of the pathogenesis of autoimmune conditions of chronic inflammatory diseases [73]. Recent scientific research has found vitamin D and, specifically, its hormonally active metabolite calcitriol to be an instrumental immunoregulatory molecule in this network [74]. Calcitriol controls various functional capabilities of T-lymphocytes, B-lymphocytes, and the cytokine milieu that interact to coordinate adaptive immune responses. In T cell regulation, calcitriol modulates antigen-presenting cellular activities so that IL-12 and IL-23 secretion is inhibited, which, in turn, suppresses the polarization of naive CD4+ T cells against pro-inflammatory Th1 and Th17 phenotypes [75]. Vitamin D modulates adaptive immunity by suppressing Th1 and Th17 differentiation while promoting Th2 responses and regulatory T cells. This shift reduces pro-inflammatory cytokines (IL-6, IL-17, TNF-α) and enhances anti-inflammatory mediators (IL-10, TGF-β), restoring immune tolerance without inducing global immunosuppression [76]. Vitamin D also inhibits B-cell proliferation, plasma cell differentiation, and autoantibody production, mechanisms particularly relevant to antibody-mediated autoimmune diseases such as SLE [77]. Such subsets have proven to be the sources of autoimmune pathogenesis and include multiple sclerosis, rheumatoid arthritis, and inflammatory bowel disease. In its turn, vitamin D enhances a functional shift to Th2 and regulatory T cells (Tregs), which is manifested by the augmented synthesis of IL-10 and IL-4 [78]. This immunological reprogramming supports immune tolerance and immunomodulates tissue-destructive inflammation, which gives mechanistic explanations of the salutary relationships between adequate vitamin D status and decreased autoimmune burden [15]. This way, vitamin D functions as a biological rheostat, balancing the negative and positive branches of the adaptive immune response.

Vitamin D has a significant effect on the functional hematology of B cells, tuning their proliferative ability, their effector activities, and their cross communication with T cells [79]. It is experimentally proven that calcitriol can slow the clonal growth of B cells, prevent the differentiation of plasma cells, and inhibit antibody production [80]. Such effects are specifically applicable in systemic lupus erythematosus (SLE), in which the hyperactive immune complex is generated, and subsequent deposition of immune complexes maintains the presence of chronic inflammation and organ destruction [81]. Vitamin D suppresses the production of antibodies to allow the body to generate protective humoral immune responses toward the pathogen and prevent the production of undesirable autoantibodies [82]. This homeostatic response is further supported by an increase in the regulatory T cell compartment via a vitamin D-mediated increase in the compartment that has an effect of synchronizing immune activity [83]. All these processes together provide a well-regulated immune environment, prevent the risk of harmful autoimmune responses, and leave the immune system intact, without a globally suppressing effect on protective immunity [84].

Vitamin D is also known to contribute to another important aspect of the immune system, which is fine-tuning of the cytokine system, a form of cellular communication [85]. Calcitriol also reduces the release of pro-inflammatory cytokines that play a role in chronic inflammation and autoimmune diseases, such as IL-6, IL-17, and tumor necrosis factor-α (TNF-α) [86]. At the same time, vitamin D increases the synthesis of anti-inflammatory cytokines, including transforming growth factor-β (TGF-β) and IL-10, thus suppressing hyper-immune responses and enhancing tissue repair mechanisms [87]. This balance mechanism of cytokines is critical in maintaining that inflammatory pathways are silent at the right time when they are not required, but can be activated in response to infectious agents [88].

These complex implications work together to show that vitamin D is more than just the immune system’s on/off switch. Rather, it functions as a sophisticated coordinator that synchronizes cytokine signaling, B-cell activity, and T-cell development. Preventing autoimmune disease, managing chronic inflammation, and maintaining the long-term functional stability of the immune defense system all depend on this intricate balance [89].

### 3.3. Vitamin D in Autoimmune Diseases

Vitamin D has some unique characteristics that can regulate the innate and adaptive elements of the immune system, making it a point of interest in the analysis of autoimmune diseases [82,90]. It can be perhaps best seen in multiple sclerosis (MS), whereby vitamin D supplements the immunosurveillance of the central nervous system by the immune system through the higher activity of regulatory T cells, Tregs, and limits the pathogenicity of autoreactive T cells on the host tissue [91]. Lots of epidemiological evidence has been able to reinforce this association; many observational studies coherently show that a lower serum 25-hydroxyvitamin [25(OH)D] level increases the chances of achieving MS and the chances of relapse in MS [92]. Geographic patterns also support the association: the incidence of MS increases in areas where there is limited sunlight coverage, and this fact makes vitamin deficiency an insufficient cofactor [93,94]. Interventional trials have sought to transfer these findings into clinical advantage; supplementation prototypes detail minor decreases in the rate of new MRI lesions and in inflammatory biomarkers, yet consequences differ among enquires [95,96]. However, the translation into meaningful clinical outcomes, including slowed disability progression, has been sporadic, highlighting the potential of vitamin D as a therapy supplement in the management of multiple sclerosis [97]. The aforementioned observations demonstrate the need to conduct large-scale and long-term randomized controlled trials to outline the best dosing schedules and individual reactions.

In addition to MS, type 1 diabetes mellitus (T1DM) is one of the conditions that has gained special interest in vitamin D studies. The hormone has a direct impact on the pancreatic β-cell viability and regulates the functionality of autoreactive T lymphocytes involved in the destruction of β-cells [98,99]. Mechanistic studies indicate that the activity of vitamin D to calcitriol, which is the hormonally active form, is not only immunosuppressive but also alters the immune milieu towards a more tolerogenic Th2/Treg instead of a proinflammatory Th1/Th17 phenotype [90]. Prospective cohort studies show good evidence in the fact that sufficient vitamin D status in early age, especially in childhood, is negatively linked to the probability of developing T1DM [100,101]. This has led to the formation of hypotheses that supplementation can play a preventive role. The clinical trial evidence is, nevertheless, still inconclusive; randomized controlled trials indicate the positive effects of clinical trials in slowing or reversing the development of T1DM, but others do not provide significant changes in the occurrence and/or progression of the disease [102]. The differences may also be due to different study designs, genetic predisposition of those who were subjected to the study, prior level of vitamin D in the body, and the time of supplementary administration. In turn, vitamin D is still considered to have potential as a T1DM risk modifier, yet its ultimate contribution to risk prevention or disease control has not been determined, and it should be subjected to more specific clinical research [103]. Table 1 demonstrates the autoimmune diseases and the mechanism of their interaction with vitamin D. Epidemiological studies consistently associate low serum 25(OH)D levels with increased risk and disease activity in MS, T1DM, RA, and SLE. Geographic patterns of MS incidence reflect not only sunlight exposure but also gene-environment interactions, supported by emerging genetic and ancient DNA evidence [104]. Interventional trials evaluating vitamin D supplementation in autoimmune diseases have yielded mixed results. Modest improvements in inflammatory biomarkers or imaging outcomes are reported in some studies, whereas effects on clinical endpoints such as disability progression or disease remission remain inconsistent [105]. These discrepancies are largely attributable to heterogeneity in baseline vitamin D status, disease stage, genetic susceptibility, dosing regimens, and concomitant therapies. Overall, current evidence supports vitamin D as an adjunctive, context-specific modulator rather than a standalone therapy in autoimmune diseases [106].

**Figure 1:**
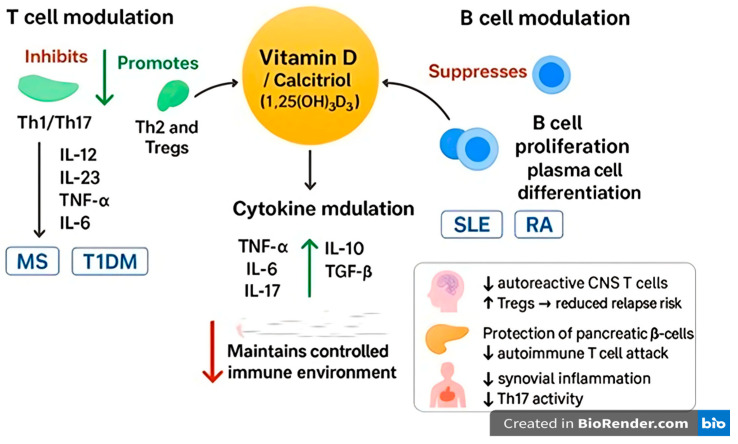
Central role of vitamin D, specifically its active ligand form, calcitriol (1,25-dihydroxyvitamin D_3_), as a molecular regulator that integrates endocrine signaling with immune modulation to maintain immune tolerance and prevent pathological autoimmunity. (Made by author Sandesh Shende with Biorender).

The immunomodulatory effects of vitamin D are also applicable to systemic autoimmune diseases, e.g., rheumatoid arthritis (RA) and systemic lupus erythematosus (SLE) [107,108]. In RA, vitamin D suppresses synovial inflammation mainly because it operates by suppressing Th17 cells and inhibiting pro-inflammatory cytokines, such as tumor necrosis factor-alpha TNF-α and IL-6, the two of which mediate joint destruction and disease progression [109]. In autoimmune diseases such as multiple sclerosis, rheumatoid arthritis, systemic lupus erythematosus, and type 1 diabetes, vitamin D deficiency is frequently observed [110]. Interventional trials have produced mixed findings, reflecting heterogeneity in baseline vitamin D status, disease stage, genetic susceptibility, and dosing regimens. Geographical associations with disease incidence now explicitly acknowledge gene-environment interactions rather than sunlight exposure alone [111]. Figure 1 illustrates the autoimmune pathology of vitamin D immunomodulation through the endocrine system. It is always shown that observational studies indicate that low levels of vitamin D correlate with an increase in the disease activity score, severity, and worse functional outcomes [112]. Clinical trials of supplementation have shown positive effects on inflammatory biomarkers and, in a few cases, small clinical effects; however, the clinical usefulness of vitamin D in RA is still disputable because of inter-study variability [105]. The association is the same in SLE but is uniquely complicated by the disease-specific factors like photosensitivity and renal dysfunction, which lead to a high occurrence of vitamin D deficiency in patients [113]. Mechanically, vitamin D inhibits B-cell autoantibody production and T reg induction, which improves immune tolerance, thus handling major immune malfunctions in SLE [114]. Clinical trials prove that supplementation can improve fatigue and serological parameters, such as the anti-dsDNA antibody levels; however, the effect of supplementation on the general disease activity is still inconsistent [115]. Taken together, these results suggest that although vitamin inadequacy is endemic in autoimmune diseases and supports a thorough biological basis of its functioning, the clinical efficacy of supplementation as a treatment option is inconsistent [116]. The data is directed towards suggesting that vitamin D has a context-specific use, and that utility is probably highest in preventive measures, restoration of deficiency, and as an addition to standard immunomodulatory treatments, but not as a standalone agent [117,118].

**Table 1:** Autoimmune diseases and their proposed mechanism of vitamin D action.

Autoimmune Disease	Proposed Mechanism of Vitamin D Action	Key Evidence	Type of Evidence	Key Study Designs	Clinical Strengths/Limitations
Multiple Sclerosis (MS) [119]	Inhibits autoreactive Th1/Th17 cells Promotes regulatory T cells (Tregs) Modulates CNS immune surveillance	Low serum 25(OH)D associated with increased MS risk and relapse rates; supplementation linked to modest reductions in MRI lesion activity	Observational, interventional	Cohort studies, case–control studies, small to moderate RCTs	Strong biological plausibility; consistent epidemiological associations; clinical outcomes inconsistent, limited effect on disability progression; heterogeneity in dosing, baseline deficiency, and genetic susceptibility
Type 1 Diabetes Mellitus (T1DM) [120]	Protects pancreatic β-cell viability Suppresses autoreactive T cells responses Promotes Th2/Treg immune balance	Childhood vitamin D sufficiency linked to reduced T1DM risk; some trials suggest delayed onset or reduced progression	Observational, interventional	Birth-cohort studies, prospective cohorts, pilot RCTs	Strong preventive signal in early life; RCT efficacy mixed; outcomes influenced by timing of exposure, baseline status, and genetic predisposition
Rheumatoid Arthritis (RA) [109]	Suppresses Th17 differentiation Reduces pro-inflammatory cytokines (TNF-α, IL-6) Dampens synovial inflammation	Low vitamin D correlates with higher disease activity scores; supplementation improves inflammatory markers in some studies	Observational, interventional	Cross-sectional studies, RCTs, and meta-analyses	Biomarker improvements documented; clinical symptom improvement inconsistent; heterogeneity in trial design, disease stage, and concurrent DMARD therapy
Systemic Lupus Erythematosus (SLE) [121]	Inhibits autoantibody-producing B cells Enhances Treg-mediated immune tolerance	High prevalence of deficiency due to photosensitivity and renal dysfunction; supplementation improves fatigue and serological markers	Observational, interventional	Cohort studies, small RCTs	High biological and clinical relevance; effects on global disease activity remain inconsistent; optimal dosing strategies not established

### 3.4. Vitamin D in Infectious Diseases

The use of sunlight and cod-liver oil as supplemental treatments for tuberculosis dates back to the pre-antibiotic era, so vitamin D’s role in infectious diseases is not a recent development [122]. Modern immunology studies have elucidated the mechanistic basis, especially the capacity of vitamin D to amplify the macrophage antimicrobial activity. The antimicrobial peptides (cathelicidin and defensins) triggered by the nutrient by calcitriol binding to the VDR enhance phagolysosome fusion and pathogen clearance [36]. The mechanism is especially beneficial to Mycobacterium tuberculosis, a pathogen that is highly able to escape intracellular killing [123]. Clinical trials and systematic reviews substantiate the hypothesis of vitamin D being complemented by increasing the speed of sputum smear conversion, showing an actual impact of the supplement on bacterial elimination [124]. However, its effects on more long-term outcomes, including relapse prevention, mortality decrease, or radiographic enrichment, are inconsistent across studies. This heterogeneity can probably be due to differences in baseline VDR levels of vitamin D, dosage schedules, and genetic variants of the VDR or vitamin D-metabolizing enzymes. Nevertheless, the gathered evidence places vitamin D as an immuno-adjuvant in the treatment of TB to add to the routine anti-TB treatment, but not to act as a specific therapeutic agent [125]. Vitamin D contributes to host defense against tuberculosis and acute respiratory infections through antimicrobial peptide induction and immune modulation. Updated meta-analyses indicate that benefits are most evident in individuals with severe deficiency and in younger populations [51].

In addition to tuberculosis, vitamin D has also been widely explored with respect to acute respiratory tract infections (ARIs), including influenza and viral pneumonias [51]. Epidemiological evidence indicates that people whose levels of 25(OH)D are lower have a high susceptibility to respiratory infections [126,127]. Mechanistically, vitamin D strengthens the defenses of mucosal barriers, maintains a normal level of cytokine production to avoid undue inflammation, and supports innate immunity by means of producing antimicrobial peptides [128]. Big, randomized controlled trials and pooled meta-analyses indicate that the protective benefit of supplementation concentrates in patients who have a deep underlying deficiency (<25 nmol/L) [129]. Notably, the route of administration does not seem to be insignificant: regular (daily or weekly) dosage is much more effective in reducing the risk, and infrequent high-dose boluses do not demonstrate such effectiveness or tend towards neutrality [26]. As a result, biomimicry—that is, physiological boluses rather than pharmacologic ones—has caused a paradigm shift in dosage regimens. Despite the clinical variability, most people agree that maintaining adequate levels of vitamin D in high-risk groups is beneficial for the general population due to the slight but significant reduction in the incidence and severity of ARI. Table 2 demonstrates the proposed mechanisms of vitamin D action in infectious diseases.

**Table 2:** Infectious diseases and proposed mechanisms of vitamin D action.

Infectious Disease	Proposed Mechanism of Vitamin D Action	Key Evidence	Type of Evidence	Key Study Designs	Clinical Strengths/Limitations
**Tuberculosis (TB)** [130]	Activates macrophage antimicrobial pathways Upregulates cathelicidin and defensins via VDR Enhances phagolysosomal fusion and intracellular killing	Historical use of sunlight and cod-liver oil; RCTs demonstrate faster sputum smear conversion	Mechanistic, interventional	In vitro studies, observational cohorts, RCTs	Strong mechanistic support; benefit as adjunct therapy; no consistent effect on relapse, mortality, or radiographic outcomes; response varies by baseline deficiency and VDR polymorphisms
**Acute Respiratory Infections (ARIs)** [51]	Strengthens mucosal barrier integrity Enhances antimicrobial peptide production Modulates cytokine responses	Low 25(OH)D linked with higher ARI susceptibility; meta-analyses show benefit mainly in severe deficiency	Observational, interventional	Large RCTs, pooled meta-analyses	Clear preventive benefit in deficient populations; bolus high-dose regimens ineffective; limited benefit in vitamin-D-replete individuals
**COVID-19 (SARS-CoV-2)** [131]	Reduces IL-6 and TNF-α–mediated hyper-inflammation Enhances innate antiviral immunity Maintains epithelial barrier function	Low vitamin D associated with severe disease and ICU admission; mixed results in supplementation trials	Observational, interventional	Cohort studies, RCTs, meta-analyses	Strong observational associations; RCT results heterogeneous; efficacy depends on baseline deficiency, timing of supplementation, and host genetics

The interest in the dual anti-inflammatory and anti-infective properties of vitamin D was also enhanced by the COVID-19 pandemic [132]. The fast associations observed through the use of observational studies were that vitamin deficiency was associated with a greater risk of infection due to SARS-CoV-2 and poorer outcomes, such as ICU admission and mortality [133]. Vitamin D has long been recognized for its role in host defense against infections. Mechanistically, calcitriol enhances macrophage antimicrobial activity, promotes phagolysosomal fusion, and induces antimicrobial peptides. In tuberculosis and acute respiratory infections, randomized trials and meta-analyses demonstrate modest protective effects, particularly in individuals with severe vitamin D deficiency and when supplementation is administered regularly rather than as high-dose boluses [62]. During the COVID-19 pandemic, observational studies reported associations between deficiency and severe disease; however, randomized trials yielded heterogeneous results, emphasizing the importance of baseline status, timing, and host genetics [134,135]. Put forward are the attenuation of the cytokine storm-modulating IL-6 and TNF-α pathways, epithelial integrity, and enhancement of antiviral innate immunity. However, the interventional studies showed mixed results. Although there have been reports of decreased ICU admissions, inflammatory markers, and mortality rates with supplementation in some RCTs and cohort trials, there have been reports of no statistically significant effect [136]. The differences may be attributed to the fact that the baseline level of vitamin D was different, the intervention used was preventive or therapeutic, the dose of vitamin D was varied, and the host genetics [137]. According to meta-analyses, prophylaxis supplementation, especially among the deficient patients, has the potential to reduce the risk of severe disease, but late supplement intake during advanced infection has limited value. Although a unanimous opinion on the topic has still not been reached, the pandemic supported the idea of vitamin D as an immuno-endocrine glucocorticoid having the potential role in viral immunity [138,139]. Most importantly, it pointed out the need to be more exact with the use of precision medicine strategies, including the baseline deficiency measures, Asian strategies, and genetic influences, to find out who is most likely to respond to supplementation.

## 4. Discussion

The present narrative review highlights vitamin D as a central immuno-endocrine regulator whose biological functions extend well beyond classical calcium–phosphate homeostasis. At the mechanistic level, calcitriol–VDR signaling exerts coordinated effects on innate and adaptive immunity by suppressing pathogenic Th1/Th17 responses, promoting regulatory T-cell differentiation, modulating B-cell autoantibody production, and enhancing antimicrobial peptide synthesis [47]. These molecular actions provide strong biological plausibility for the observed associations between vitamin D deficiency and increased susceptibility to autoimmune and infectious diseases. However, despite consistent mechanistic and epidemiological support, interventional trials evaluating vitamin D supplementation have yielded mixed and often inconsistent clinical outcomes, underscoring the complexity of translating immunological plausibility into therapeutic efficacy [41].

A major contributor to this inconsistency is the substantial heterogeneity across clinical trials. Variability in baseline vitamin D status is particularly influential, as benefits appear largely confined to individuals with documented deficiency, while supplementation in vitamin-D-replete populations often yields minimal or no clinical effect [140]. In addition, differences in dosing regimens, including daily or weekly physiological supplementation versus intermittent high-dose bolus administration, have produced divergent outcomes, with accumulating evidence favoring sustained low-dose strategies. Genetic polymorphisms in VDR and vitamin D-metabolizing enzymes (CYP2R1, CYP27B1, GC) further modulate individual responsiveness, contributing to inter-individual variability in immune and clinical responses [141,142]. Disease-specific factors, such as stage of autoimmunity, inflammatory burden, concomitant immunomodulatory therapies, and selected clinical endpoints, further compound trial heterogeneity and limit direct comparability across studies [143].

Importantly, emerging data suggest that vitamin D does not act in isolation within immune regulatory networks. Interactions between vitamin D and other immuno-logically relevant micronutrients, particularly zinc and omega-3 fatty acids, may influence immune cell signaling, cytokine resolution, and inflammatory homeostasis, as demonstrated in large randomized trials [144]. This reinforces the concept that vitamin D should be considered within a broader framework of immunonutrition rather than as a standalone immunotherapeutic agent. Furthermore, observational associations must be interpreted cautiously, as low circulating vitamin D levels may reflect underlying inflammation, chronic illness, or reduced sunlight exposure rather than direct causality [145]. Collectively, these findings emphasize the need for precision-based, stratified randomized controlled trials that incorporate baseline vitamin D status, genetic susceptibility, dosing strategy, disease phenotype, and co-nutrient interactions to clarify the true clinical utility of vitamin D in autoimmune and infectious diseases [146].

The discussion centers on vitamin D’s role in immuno-endocrine regulation, which definitely expands beyond its traditional roles in calcium-phosphate homeostasis and bone maintenance [147]. VDR, the activation of the immune and endocrine cells coordinates a comprehensive set of transcriptional and epigenetic activities that regulate host defense, immune tolerance, and inflammatory balance [148]. On the molecular checkpoint level, vitamin D modifies Tucker helper cell polarization, biases the growth of regulatory T (Treg) cells, suppresses the activity of autoreactive B cells, regulates dendritic-cell development, induces the generation of antimicrobial peptides, and increases phagocytic capabilities of macrophages [149]. Together, these processes contribute to the ability of vitamin D combinational immunological biomarkers to be at the center of both endocrinology and immunology, creating significant implications in infectious disease defense and pathogenesis of autoimmunity [41].

Deficiency of vitamin D has been consistently related to increased vulnerability and escalated dilemma in a list of autoimmune ailments such as multiple Sclerosis, type 1 diabetes mellitus, rheumatoid arthritis, and systemic lupus erythematosus [150]. There is mechanistic evidence supporting such associations, which underscores the ability of vitamin D to suppress Th1/Th17 responses, increase regulatory T cells, and inhibit B-cell activation [151]. However, interventional trials have had mixed findings, with some demonstrating supporting effects of reduced MRI lesion activity in MS or prevention of inflammatory biomarkers in RA, and others demonstrating insignificant clinical effect [152]. Such discrepancies suggest that treatment response is dependent on preexisting vitamin D levels, host genetic differences in the VDR or the various metabolic enzymes, and the duration of the treatment provided regarding disease development [153,154]. Cases of experiments based on parallels in the context of infectious diseases-tuberculosis, lower respiratory tract infections, and COVID-19 have shown strong mechanistic plausibility, increased macrophage antimicrobial activities, and decreased susceptibility of infection among subjects with deficiencies but have produced mixed clinical trial results [155]. Most importantly, long-term daily or weekly interventions have been demonstrated to have apparent protective effects in deficient cohorts, but bolus schedules or vitamin D supplementation in individuals with sufficient vitamin D do not, as a rule, bring about benefits [156].

These discrepancies point to ongoing disagreements in the field, first and foremost, about the most efficient method of administration. Many researchers advocate for daily low-dose supplementation rather than sporadic high-dose injections because they are concerned that supraphysiologic dosage boluses may disrupt systemic homeostasis or only have a transient effect [157]. Further, the variability between individuals in physiological response would suggest that there is a need to take into account individual dosing thresholds; existing data suggests a non-linear, threshold effect, which is restricted to individuals proving deficient. Therefore, the shift to the risk-stratified approach to identifying vulnerable populations means that risk may have to be employed by the public-health initiatives instead of the blanket supplementation interventions [158]. Host genetics is a third important factor to take into account. Certain polymorphisms of VDR, CYP2R1, and GC significantly affect individual responsiveness, which calls for the development of precision-medicine strategies utilizing genomic, biochemical, and immunological assay technologies that combine genomic and biochemical with immunological data [159]. Lastly, low circulating vitamin D may be a stand-in for underlying illness, systemic inflammation, or lack of sun exposure rather than a cause, making observational studies susceptible to confounding. To more confidently identify causal pathways, rigorous techniques like Mendelian randomization, well-stratified randomized control trials, and mechanistic studies are required [160].

Altogether, the sum of the evidence outlines vitamin D as a compulsory mediator in the immuno-endocrine wiring with a significant treatment potential [140]. However, basing clinical translation on inter-trial heterogeneity, confounding variables, and lack of concurring dosing protocols continue to pose impediments to the road. Subsequent studies must therefore focus on stratified randomized controlled trials on nutritionally deficient groups, incorporate serial immune-phenotyping due to the need to monitor predisposition of the biomarker markers over time, and consider host genetic susceptibility that influences the response [161]. Additionally, this arsenal of therapeutic tools may continue to be expanded by the development of new vitamin D analogs that maintain immunomodulatory criteria and the depressive capacity of hypercalcemia, in addition to the investigation of synergistic changes in the interaction between vitamins and micronutrients, including zinc and omega-3 fatty acids [162]. This review positions vitamin D at the intersection of immunology and endocrinology, with robust mechanistic plausibility but variable clinical efficacy. Trial heterogeneity arises from differences in baseline vitamin D levels, dosing strategies, genetic polymorphisms, disease phenotype, and outcome selection. Emerging evidence suggests synergistic interactions between vitamin D and micronutrients such as zinc and omega-3 fatty acids, reinforcing the concept of integrated immunonutrition rather than isolated supplementation [163]. Precision-medicine approaches incorporating genetic, biochemical, and immunological profiling are essential to optimize clinical translation [164]. Finally, vitamin D should be previously accepted not as immunotherapy but as a situational modulator whose possible advantages in clinical application depend on the specifics of biology, the context of the environment, and the stage of disease [89]. The adoption of this subtle framework could help answer most of the remaining controversy and better establish vitamin D in the new field of precise immunology.

It turns out that vitamin D can function as a circumstellar-dependent regulator whose effectiveness is imposed upon host biology, exposure to environmental factors, and disease stage, but it cannot be considered a universal immunotherapeutic agent. Taking this stance can help resolve the majority of the current debate and improve our understanding of vitamin D’s function in precise immunology. This review highlights vitamin D as an immuno-endocrine regulator with mechanistic plausibility but variable clinical efficacy. Heterogeneity across trials arises from baseline vitamin D levels, dosing regimens, genetic polymorphisms, disease phenotype, and outcome selection. Emerging evidence supports synergistic interactions between vitamin D and micronutrients such as zinc and omega-3 fatty acids, reinforcing the concept of integrated immunonutrition rather than isolated supplementation.

## 5. Conclusions

Vitamin D should be viewed as a situational and context-dependent immunomodulator rather than a universal therapy. Its clinical utility is greatest when deficiency is corrected and when applied as an adjunct to standard care. Future stratified trials incorporating genetics, baseline status, and co-nutrient interactions are essential for precision immunology applications.

## Data Availability

Not applicable.
